# Evolutionary Divergence of the Novel Staphylococcal Species *Staphylococcus argenteus*

**DOI:** 10.3389/fmicb.2021.769642

**Published:** 2021-11-19

**Authors:** Shi Wu, Rui Pang, Jiahui Huang, Feng Zhang, Zhihe Cai, Jumei Zhang, Moutong Chen, Liang Xue, Qihui Gu, Juan Wang, Yu Ding, Qiang Wan, Qingping Wu

**Affiliations:** ^1^Guangdong Provincial Key Laboratory of Microbial Safety and Health, State Key Laboratory of Applied Microbiology Southern China, Institute of Microbiology, Guangdong Academy of Sciences, Guangzhou, China; ^2^Guangdong Huankai Microbial Science and Technology Co. Ltd., Guangzhou, China

**Keywords:** *Staphylococcus argenteus*, population structure, pangenome, genetic divergence, regional specialization

## Abstract

Currently, invasive infections caused by *Staphylococcus argenteus*, which is a recently named staphylococcal species, are increasingly reported worldwide. However, only a few genomic studies of *S*. *argenteus* have offered comprehensive information regarding its genetic diversity, epidemiological characteristics, antimicrobial resistance genes (ARGs), virulence genes and other profiles. Here, we describe a comparative genomic analysis by population structure, pangenome, panmobilome, region-specific accessory genes confer an adaptive advantage in 153 *S*. *argenteus* strains which comprised 24 strains sequenced in this study and 129 strains whose genome sequences were available from GenBank. As a result, the population of *S*. *argenteus* comprised seven genetically distinct clades, including two major clades (C1 and C2), with distinct isolation source patterns. Pangenome analysis revealed that *S*. *argenteus* has an open pangenome composed of 7,319 genes and a core genome composed of 1,508 genes. We further determined the distributions of 75 virulence factors (VFs) and 30 known ARGs and identified at least four types of plasmids and 93 complete or partial putative prophages. It indicate that *S*. *argenteus* may show a similar level of pathogenicity to that of *S*. *aureus*. This study also provides insights into the evolutionary divergence of this pathogen, indicating that the geographical distribution was a potential driving force behind the evolutionary divergence of *S*. *argenteus*. The preferential horizontal acquisition of particular elements, such as staphylococcal cassette chromosome *mec* elements and plasmids, was observed in specific regions, revealing potential gene exchange between *S*. *argenteus* strains and local *S*. *aureus* strains. Moreover, multiple specific genes related to environmental adaptation were identified in strains isolated from East Asia. However, these findings may help promote our understanding of the evolutionary divergence of this bacterium at a high genetic resolution by providing insights into the epidemiology of *S*. *argenteus* and may help combat its spread.

## Introduction

Recently, a novel coagulase-positive staphylococcal species, *Staphylococcus argenteus*, was distinguished from on-pigmented *Staphylococcus aureus* (Holt et al., 2011; Tong et al., 2015). It was first identified as *S*. *aureus* according to its phenotype; however, according to whole-genome sequence analysis, the two species exhibit identical 16S rRNA genes but are distinct in terms of average nucleotide identity (ANI) (87%) and digital DNA–DNA hybridization results (34%) (Ng et al., 2009; Ruimy et al., 2009; Tong et al., 2015). *Staphylococcus argenteus* was first found in northern Australia in 2002 (McDonald et al., 2006). Since then, several studies have reported sporadic cases of *S*. *argenteus* infections, mainly in Sweden, Denmark, France, Belgium, China, Japan, New Zealand, Fiji, Cambodia, French Guiana, and Trinidad and Tobago (Ruimy et al., 2009, 2010; Jenney et al., 2014; Monecke et al., 2014; Ritchie et al., 2014; Dupieux et al., 2015; Thaipadungpanit et al., 2015; Chantratita et al., 2016; Hansen et al., 2017; Schuster et al., 2017; Jiang et al., 2018; Olatimehin et al., 2018; Tång Hallbäck et al., 2018; Kitagawa et al., 2020), which confirmed that this species is globally distributed. Based on multilocus sequence typing (MLST), *S*. *argenteus* is easy to identified. Theoretically, several *arcC* alleles (36, 151, 207, and 272) and *pta* alleles (39, 107, 145, 175, 198, 256, 268, and 287) clustered in a single branch containing known *S*. *argenteus* isolates and were not shared with *S*. *aureus* (Thaipadungpanit et al., 2015). Clonal complex 75 (CC75), CC2250, CC1223, CC2485, and CC2198 were the common sequence types (STs) found in this species, most of which have been previously reported as causes of community infections (Chantratita et al., 2016).

Initially, *S*. *argenteus* was thought to be less virulent than *S*. *aureus* due to its lack of staphyloxanthin, but subsequent deaths have shown its clinical importance (Thaipadungpanit et al., 2015; Chantratita et al., 2016; Chen et al., 2018). Moreover, genes encoding toxins such as staphylococcal enterotoxin, hemolysin, and Panton-Valentine leukocidin have been associated with *S*. *argenteus* (Zhang et al., 2017). A recent study indicated the ability of *S*. *argenteus* to cause staphylococcal food poisoning, revealing that this bacterium is highly toxigenic and is also a potential foodborne pathogen (Wakabayashi et al., 2018). Thus, it is important to understand the genetic factors that allow some strains to spread aggressively, whereas others exist asymptomatically.

The epidemiology of *S*. *argenteus* and the phenotypic diversity present among different strains are reflected in their genotypes. Unfortunately, classical genotyping methods, such as pulsed-field gel electrophoresis (PFGE) or MLST, rely on the evaluation of highly conserved housekeeping genes that are representative of the vertical gene pool and thus do not provide a sufficient resolution for the prediction of disease phenotypes (Bosi et al., 2016). Therefore, it may be possible to analyse multiple *S*. *argenteus* genomes to discover factors that could be predictors of disease phenotypes and virulence capabilities. This approach to comparative genomics analysis might be more effective for characterizing the breadth of the gene repertoire accessible to individual microbes and understanding the amount of additional genomic data required for the proper characterization of this repertoire (Tettelin et al., 2008). The definition of the pangenome of a bacterium sheds light on its biology and lifestyle and has implications for the definition of the species itself. Currently, more than one hundred *S*. *argenteus* genome sequences are available in the National Center for Biotechnology Information (NCBI) Genome database. In addition, we obtained multiple *S*. *argenteus* strains in our previous work (Wu et al., 2020). In the present study, we intended to construct a comparative genomic analysis of the *S*. *argenteus* species and provide insight into the evolutionary divergence of this bacterium that represents the breadth of its genetic, phenotypic, and geographical characteristics.

## Results

### Population Structure of *Staphylococcus argenteus*

**FIGURE 1:**
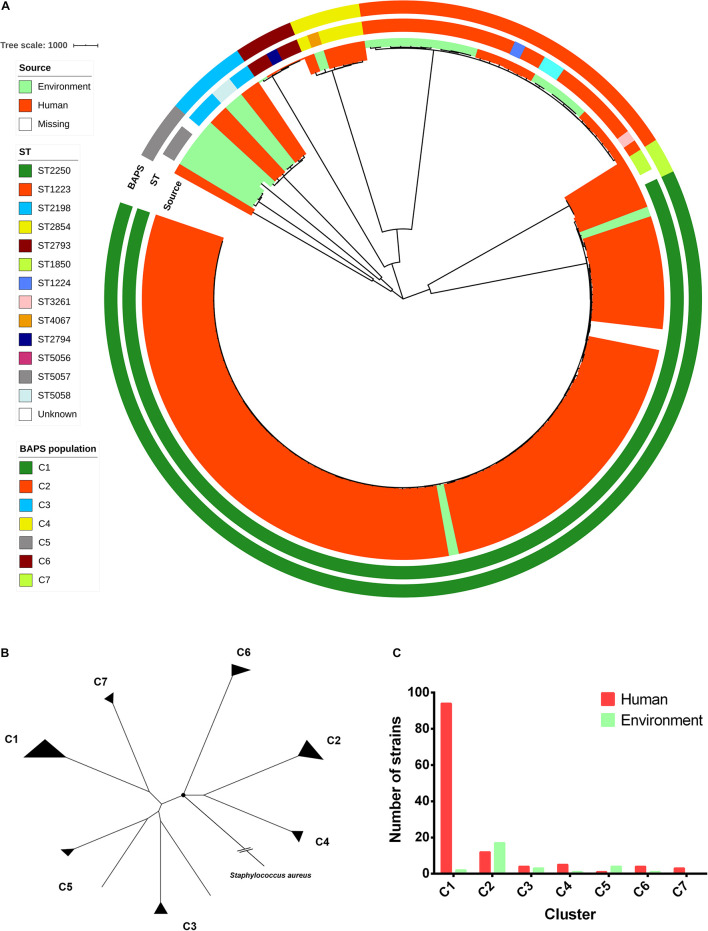
Phylogenomic and population structure of *Staphylococcus argenteus*. **(A)** Maximum-likelihood (ML) tree of *S*. *argenteus* constructed from 37,309 core genome single-nucleotide polymorphisms (SNPs). The clade colors indicate the source of each strain. The inner bar color indicates the sequence type of each strain, and the outer bar color indicates the Bayesian analysis of population structure (BAPS) cluster to which each strain is attributed. **(B)** ML tree reconstructed from the core genome SNPs using *S*. *aureus* as an outgroup. The black circle indicates the root of the tree. **(C)** The number of non-clonal human and environmental strains in each cluster.

The set of *S*. *argenteus* samples comprised 24 strains sequenced in this study and 129 strains whose genome sequences were available from GenBank (Supplementary Table 1). For these strains, except 2 strains (F87619 and M21126) were missing the source information, 123 strains were belonged to human and 28 strains were belonged to non-human (food and animal) from 13 countries (China, Singapore, Malaysia, Thailand, Fiji, Israel, Australia, United States, France, Sweden, Denmark, Germany, and Africa). Most strains were collected from 2010 to 2016 (Supplementary Table 1). Overall, the *S*. *argenteus* population comprised 14 STs, most of which were assigned to ST2250 (98/153, 64%) and ST1223 (25/153, 16%). Among all of these strains, 37,309 core genome single-nucleotide polymorphisms (SNPs) were identified. The concatenated SNPs were used to reconstruct a maximum-likelihood (ML) phylogenetic tree of *S*. *argenteus* (Figure 1A). The phylogeny revealed high genetic diversity between different STs of *S*. *argenteus*. The genetic population structure inferred by Bayesian analysis revealed seven genetically divergent populations that were congruent with the ST clusters (Figure 1A and Supplementary Figure 1). Additionally, some populations contained multiple STs. The divergence between clusters was also supported by the results of ANI analysis (Supplementary Figure 2). Bacterial strains from the same cluster shared ANI values of >99%, which were distinct from those of other clusters (average ANI value ∼98%). Using *S*. *aureus* as an outgroup, we found that *S*. *argenteus* clusters started branching out at a node close to C6. The two largest clusters, C1 and C2, branched in different directions from the starting node (Figure 1B).

Notably, a distinct pattern linking strain phylogeny and isolation sources was found (Figure 1A). C1 appeared to contain a dominant proportion of strains isolated from humans, while C2 was derived from multiple sources (Figure 1C and Supplementary Table 1), including humans, animals, and foods. In addition, some of the genetically related strains seemed to be spatially clustered [e.g., C1 in Southeast Asia (*P* value = 1.057 × 10^–09^, chi-square test) and C2 in East Asia (*P* value = 2.443 × 10^–09^, chi-square test)] (Supplementary Figure 3).

### *Staphylococcus argenteus* Pangenome

**FIGURE 2:**
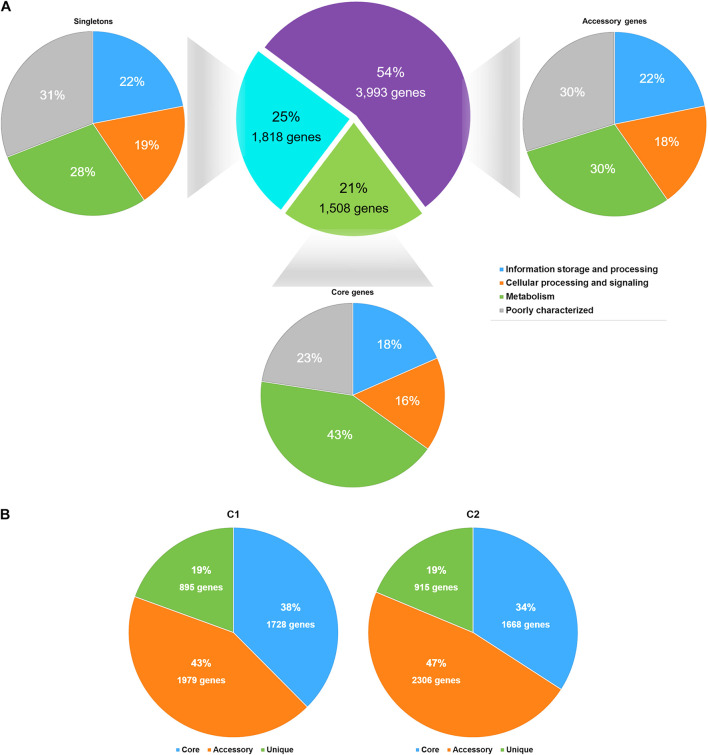
The statistics of the *S*. *argenteus* pangenome. **(A)** The *S*. *argenteus* pangenome was subdivided into three categories: (i) the core genome (the set of genes shared by all genomes), (ii) the accessory genome (the set of genes present in some but not all genomes), and (iii) the unique genome (genes that are unique to a single genome). The COG functional classification of each gene in a group is presented. **(B)** Proportions of the core genome, accessory genome, and unique genome in *S*. *argenteus* clusters C1 and C2.

The pangenome of *S*. *argenteus* consisted of 7,319 protein-coding genes, among which 1,508 genes were determined to be core genes (shared by virtually all strains), 3,993 were determined to be accessory genes (present in some but not all strains), and 1,818 were determined to be singletons (unique to individual strains) (Figure 2A and Supplementary Table 2). Overall, the phylogeny inferred from the full alignment of the core genes was in accordance with that inferred from the core genome SNPs (Supplementary Figure 4). Approximately 55% of the core genes were assigned to metabolic functions (Figure 2A), including functions related to amino acids, carbohydrates, inorganic ions, and lipid metabolism, etc.

We then compared the distributions of core genes, accessory genes, and singletons in C1 and C2. C1 and C2 showed similarly amount of core genome and singleton categories (Figure 2B). However, the proportion of accessory genes differed considerably between these two clusters, and C2 possessed more variability in the accessory genome than C1. This difference was also supported by the average genome sizes of C1 and C2. The average genome size of C1 contained 2604 protein-coding genes, among which 33% were accessory genes on average. In contrast, the genome of C2 strains contained more protein-coding genes (2,704 on average) and a larger proportion of accessory genes (37%, on average) than the C1 genome. The differential presence of accessory genes (e.g., the potential virulence factors *hysA* and *hysB*, and potential antibiotic resistance gene *msrA* presence in C2 than in C1) indicated the potential diversity of virulence and antibiotic resistance among different *S*. *argenteus* clusters.

#### Virulence Factors

**FIGURE 3:**
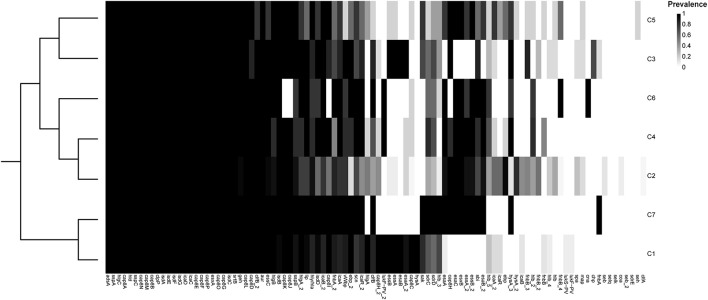
Prevalence of different virulence genes in each of the *S*. *argenteus* clusters.

Through the analysis of virulence factors (VFs), we identified a total of 75 different VFs present in at least one of the 153 *S*. *argenteus* strains (Figure 3 and Supplementary Table 3). Approximately 50% of these VF genes harbored complete coding sequences of the corresponding proteins, indicating that they are likely expressed in this bacterium. All of the core VFs were homologous to the VFs identified in *S*. *aureus*, including multiple genes related to cell adhesion properties [intercellular adhesion (*icaA*, *icaB*, *icaC*, *icaD*, and *icaR*); clumping factors (*clfA*, *clfB*, *coa*, *cna*, *sdrC*, *sdrD*, *sdrE*, *spa*, and *vWbp*); and fibronectin-binding proteins (*fnbA* and *fnb*B)], hemolysin genes (*hly/hla*, *hlb*, *hld*, *hlgA*, *hlgB*, and *hlgC*), and type VII secretion system genes (*esxA*, *esaA*, *essA*, *esaB*, *essB*, *essC*, *esaC*, and *esxB*); eleven polysaccharide capsule (PC) synthesis-related genes (*cap8A*, *cap8B*, *cap8D*, *cap8E*, *cap8F*, *cap8G*, *cap8L*, *cap8M*, *cap8N*, *cap8O*, and *cap8P*); five iron-regulated protein-coding genes (*isdA*, *isdC*, *isdE*, *isdF*, and *isdG*); and a subset of enterotoxin genes (*seb*, *seh*, *selk*, and *selq*). Moreover, major virulence regulatory systems of *S*. *aureus* (Jenul and Horswill, 2019), such as *agr* [Cell-to-cell communication (quorum sensing) with AIPs as signal; *agr* activation leads to expression of exo-toxins and exo-enzymes], *saeRS* (Induction of exo-protein production, including many virulence factors), *srrAB* (Oxygen-responsive TCS; induction of *plc* and *ica* expression; repression of *agr*, *TSST-1*, and *spa*), *arlRS* (Autolysis and cell surface TCS; induction of MgrA expression and repression of *agr* and autolysis), *sarA* (Cytoplasmic regulator; induction of exo-proteins and repression of spa), *rot* (Cytoplasmic regulator of toxins and extracellular proteases; *agr* activation prevents Rot translation), *mgrA* (Cytoplasmic regulator; induction of efflux pumps and capsule expression; repression of surface proteins), *sigB* (Stationary phase sigma factor; inhibits agr activity), were also identified in all 153 *S*. *argenteus* strains (Supplementary Table 2). Among these VFs, 27 were shared by all of the strains, forming a core set of VFs. Notably, Panton–Valentine leukocidin (PVL), which is encoded by the *lukS* and *lukF* genes, was present in only 9% of strains in C1. In addition, staphylokinase (SAK), which is a VF that promotes bacterial conversion into human plasmin (Okada et al., 2000), was absent from the C2 branches (including C4 and C6).

#### Resistance Genes

**FIGURE 4:**
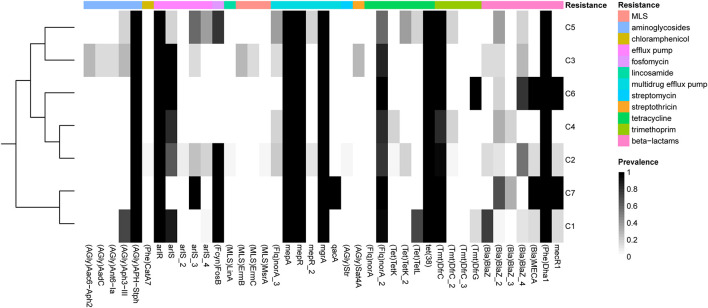
Prevalence of antibiotic resistance-related genes in each of the *S*. *argenteus* clusters. The genes conferring different types of resistance are indicated by bars of different colors.

We identified 30 known antibiotic resistance genes (ARGs) in at least one of the 153 *S*. *argenteus* strains. These ARGs might confer resistance to β-lactams (*blaZ*, *mecA*, *mecR1*, and *DHA-1*), tetracycline [*tet*(*K*), *tet*(*L*), *tet*(*A*), and *tet*(*38*)], macrolide-lincosamide-streptogramin (MLS) [*msr*(*A*), *erm*(*B*), and *erm*(*C*)], lincosamide (*linA*), aminoglycosides [*ant*(*6*)*-Ia*, *aacA-aphD*, *aph*(*3′*)*-IIIa*, *aph-stph*, and *aadC*), trimethoprim (*dfrC* and *dfrG*), streptomycin (*str*), chloramphenicol (*cat*), streptothricin (SAT-4) and fosfomycin (*fosB*), as well as antibiotic efflux pumps (*norA*, *mepA*, *mepR*, *qacA*, *arlS*, *arlR*, and *mgrA*) (Supplementary Figure 4 and Supplementary Table 4). Most of these resistance genes are encoded by plasmids, such as *ant*(*6*)*-Ia*, *blaZ*, *cat*, *dfrG*, *erm*(*B*), *erm*(*C*), *fosB*, *msr*(*A*), *str*, *tet*(*K*), and *tet*(*L*), or are associated with transposons [*aacA-aphD*, *aph*(*3′*)*-IIIa*, *erm*(*B*), and *dfrC*]. Transposon-mediated transfer has long been recognized as a key mechanism by which ARGs are disseminated in *S*. *aureus* (Partridge et al., 2018). Additionally, the multidrug efflux pumps encoded by *norA*, *mepA*, *mepR*, *arlS*, *arlR*, and *mgrA* are widely distributed in *S*. *argenteus* and *S*. *aureus*. Interestingly, most plasmid- or transposon-encoded resistance genes, such as *tet*(*K*), *cat*, *str*, *msr*(*A*), and *linA*, were absent in C1, while C2 contained most of these genes (Figure 4).

### *Staphylococcus argenteus* Panmobilome

We analyzed the panmobilome of *S*. *argenteus* genomes to identify the different types of mobile genetic elements (MGEs) harbored by this species. These MGEs might facilitate the expansion of the ecological niche of this bacterium, including the dissemination of antimicrobial resistance (AMR) and VFs within its populations.

#### Plasmids

**FIGURE 5:**
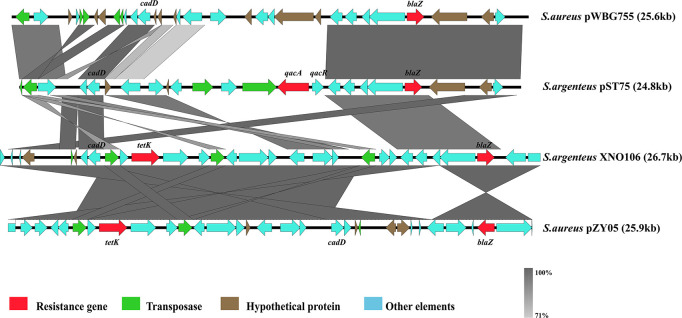
Schematic representation comparing two plasmids described in *S*. *argenteus* with other similar plasmids found in *S*. *aureus*.

At least four types of plasmids have been found in *S*. *argenteus*, all of which were reported to carry multiple antibiotic resistance-related genes (Holt et al., 2011; Moradigaravand et al., 2017). Plasmid-*tet*(*L*)-*cadD*-*blaZ* and plasmid-*cadD*-*blaZ* were previously characterized and are closely related to *S*. *aureus* plasmid p18813-P04 (Kennedy et al., 2010; Moradigaravand et al., 2017). These two plasmids were mainly present in strains isolated in Thailand (87% from Thailand 13% from other countries). Two other plasmids, plasmid-*cadD*-*tet*(*K*)-*blaZ* and plasmid-*cadD*-*qacA*-*blaZ* (pST75), were mainly identified in strains from China [90% plasmid-*cadD*-*tet*(*K*)-*blaZ* found in strains XNO106, SJTU_F20419 and SJTU_F21285, and 10% from Thailand] and Australia (pST75 found in two Australia strains MSHR1132 and NCTC13711), respectively. Notably, plasmid-*cadD*-*tet*(*K*)-*blaZ* was closely related to *S*. *aureus* plasmid pZY05, and pST75 was closely related to *S*. *aureus* plasmid pWBG755 (Figure 5). This indicates that *S*. *argenteus* strains from different geographical regions might specifically obtain plasmids homologous to those carried by their *S*. *aureus* counterparts.

#### Staphylococcal Cassette Chromosome *mec* Elements

**FIGURE 6:**
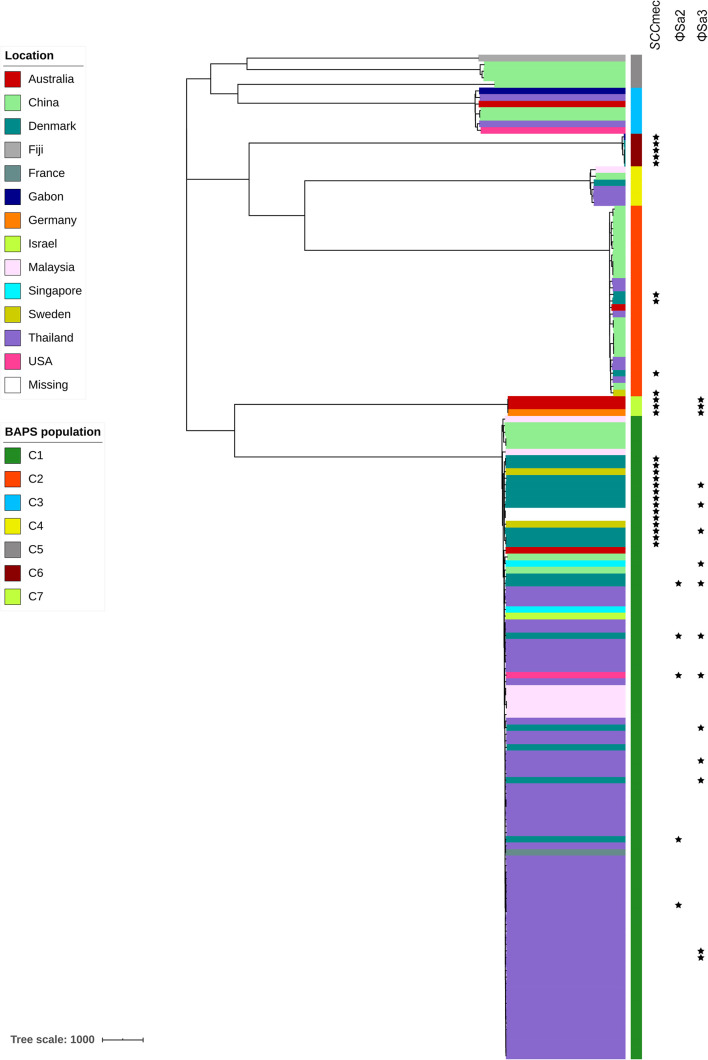
The presence of staphylococcal cassette chromosome *mec* (SCC*mec*) elements and prophages ΦSa2 and ΦSa3 among 153 *S*. *argenteus* strains. The clade colors indicate the source of each strain. The bar color indicates the BAPS cluster to which each strain is attributed. The star indicates the presence of the corresponding element.

Staphylococcal cassette chromosome *mec* (SCC*mec*) MGEs are closely related to methicillin-resistant *S*. *aureus* (MRSA). Evidence of the presence of SCC*mec* elements in *S*. *argenteus* has been reported previously (Argudín et al., 2016; Zhang et al., 2017). Here, we found that all SCC*mec* elements in *S*. *argenteus* belonged to SCC*mec* type IV. Interestingly, the distribution of the SCC*mec* elements was discordant with the genetic topology of the *S*. *argenteus* clades but was generally associated with their geographical locations of isolation (*P* value = 3.426 × 10^–16^, chi-square test; Figure 6). Strains carrying SCC*mec* elements could be found in C1, C2, C3, and C7 of *S*. *argenteus*. However, none of the strains isolated from Asia carried an SCC*mec* element. In contrast, approximately 65% of the strains isolated in Europe carried this element. This indicated that the acquisition of the SCC*mec* element in *S*. *argenteus* was a recent event and was independent of the genetic divergence of *S*. *argenteus* clades.

#### Prophages

In total, 93 complete or partial putative prophages were identified in the *S*. *argenteus* genomes associated with all clusters. The lengths of these prophage elements ranged from 1.5 to 151 kb. However, most of these elements were only present in specific clades. For example, the PVL-encoding prophage ΦSa2 was found only in several strains from C1 (Figure 6), and the SAK-encoding prophage ΦSa3 was found in all strains from C6 and some from C1 (Figure 6). There were multiple VFs linked to the *S*. *argenteus* prophages, most of which, such as *sspC* encoding staphostatin B and *seb* encoding staphylococcal enterotoxin B, were also found in the genome of *S*. *aureus*. This indicates that prophage-mediated pathogenicity is very similar between these two *Staphylococcus* species. However, the number of predicted prophages varied considerably among strains collected from different geographical regions. The strains isolated in Europe and East Asia showed significantly higher prophage numbers than those isolated from Southeast Asia and Oceania (*P* value < 1 × 10^–10^, Student’s *t*-test, Supplementary Figure 6), even among strains belonging to the same genetic clades.

### Region-Specific Accessory Genes Confer an Adaptive Advantage in *Staphylococcus argenteus*

We noted that some genomic elements of *S*. *argenteus* showed geography-related features. To provide insights into the impact of the geographical distribution on *S*. *argenteus* populations, we redefined all analyzed strains according to the regions from which they were collected (Southeast Asia, East Asia, Europe, Oceania, Africa, and North America). Strains isolated from Southeast Asia, East Asia, and Europe constituted 92% of the total strains included in our dataset; thus, we focused on the accessory genes that were only present in strains from these regions. Unexpectedly, none of the specific genes was found in the strains from Southeast Asia, and only 7 genes were specifically found in the strains from Europe, while the number of East Asia-specific genes was 256, which vastly exceeded the numbers in the other groups (Supplementary Table 5). One of the Europe-specific genes encoded a protein conferring trimethoprim resistance (*dfrG*), while the others were proteins of unknown function. Among the East Asia-specific genes, one was associated with tetracycline resistance [*tet*(*K*)], and the other four genes were annotated to the KEGG pathways of beta-lactam resistance (*mecI* and *pbp3*) and vancomycin resistance (*mraY* and *murF*) (Supplementary Figure 7). We also found that five of the East Asia-specific genes were involved in the pathway of *S*. *aureus* infection (Supplementary Figure 7), indicating a potentially high infection risk. In addition, four genes (*plc*, *secA*, *secD*, and *wecB*) were attributed to the pathways of quorum sensing and biofilm formation (Supplementary Figure 7), implying that the strains from East Asia might have a strong capacity to form biofilms. Overall, the geography-based diversity in the accessory genome of *S*. *argenteus* might be a result of regional specialization that facilitates its adaptation to specific environments.

## Discussion

As a recently named novel species, only a few genomic studies of *S*. *argenteus* have offered comprehensive information regarding its genetic diversity, epidemiological characteristics, AMR genes, virulence genes and other profiles (Zhang et al., 2017). Therefore, in the present study, we performed a large-scale genomic comparison of this species previously described as a divergent lineage of *S*. *aureus*. Accordingly, we found that *S*. *argenteus* consists of seven genetically distinct clades, the population structures of which are roughly similar to those described by Moradigaravand et al. (2017). We also revealed the existence of three new clades, among which C5 (ST5057) and C7 (ST1850) were most closely related to the previously described branch C1 (ST2250), while C6 (mainly ST2793) was most closely related to branch C2 (ST1223) (Figure 1B). However, the numbers of strains in these clades were rather limited, and we therefore obtained very little new information on the general features of these clades. Further acquisition of more *S*. *argenteus* strains or the extraction of potential *S*. *argenteus* genome sequences from an increasing number of environmental and clinical metagenomics datasets may help promote a higher-genetic resolution understanding of the evolutionary divergence of this bacterium.

Previously, *S*. *argenteus* was considered less virulent than *S*. *aureus*. On the basis of a study in Australia, it was reported that this species was associated mainly with skin and soft tissue infections but rarely with bacteremia (3/220 cases) (Tong et al., 2013). Additionally, a comparative study of 311 cases of sepsis caused by *S*. *argenteus* and *S*. *aureus* in Thailand showed a similar outcome after 28 days (Chantratita et al., 2016). Moreover, *S*. *argenteus* lacks staphyloxanthin, which is a carotenoid pigment that confers resistance against oxidative stress and neutrophil killing (Liu et al., 2005; Clauditz et al., 2006). In this study, we determined the distribution of 75 VFs in *S*. *argenteus* genomes, including multiple genes related to cell adhesion properties, hemolysin genes, type VII secretion system genes, PC synthesis-related genes, iron-regulated protein-coding genes and a subset of enterotoxin genes. These VFs play pivotal roles in host immune evasion and the infection of animals and humans (McAdow et al., 2012; Zecconi and Scali, 2013). Importantly, some specific virulence genes, such as PVL and *sak*, have also been detected in *S*. *argenteus* genome. As we know, PVL is a bacteriophage-encoded bicomponent leukotoxin that is in some strains of *S*. *aureus* and plays a key role in leukocytolysis and tissue necrosis (Shallcross et al., 2013). Many studies have reported an association between PVL genes and invasive disease, indicating that PVL is an epidemiological marker of a syndrome of severe infection (Gillet et al., 2002; Ellis et al., 2004; Lee et al., 2005; Pannaraj et al., 2006). In fact, the risk of death associated with PVL-positive *S*. *aureus* has been reported to be higher than that associated with non-PVL-producing *S*. *aureus* (Gillet et al., 2002). In addition, staphylokinase, which is encoding by *sak* gene, is a serine protease-like molecule produced by *S*. *aureus*, to interact with and neutralize α-defensins, important effector molecules of the host innate and adaptive immune system (Jin et al., 2004). As a result, staphylokinase expression may act as an important factor for bacterial persistence in susceptible groups of immunocompromised and elderly patients (Bokarewa et al., 2006). Therefore, the emergence of these virulence genes in *S*. *argenteus* is not a good phenomenon.

Moreover, prophages, which are important vectors carrying VFs in *S*. *aureus* (Feng et al., 2008), show diverse distributions in *S*. *argenteus*. As we know, *S*. *aureus* bacteriophages as important contributors to pathogenesis and the evolution of staphylococcal genomes (Novick, 2003; van Wamel et al., 2006). In this study, some of these elements were prevalent in both *S*. *aureus* and *S*. *argenteus*, such as *S*. *aureus* phage phiNM2, which revealed essential contributions to the pathogenesis of staphylococcal infection (Bae et al., 2006). By Bae et al. (2006)’s report, *S*. *aureus* phage phiNM2 were first identified in the genome of *Staphylococcus aureus* Newman, a human clinical isolate. This prophage occurs in culture and during animal infection, displayed organ specific virulence defects in a murine model of abscess formation. Thus, our findings indicate that *S*. *argenteus* may show a similar level of pathogenicity to that of *S*. *aureus*.

Pangenome analysis was performed to investigate the evolutionary differences in the overall gene contents within different *S*. *argenteus* clusters. In this study, the pangenome of *S*. *argenteus* was in accordance with a previous report in *S*. *aureus*, which also possesses an open pangenome (Bosi et al., 2016). The pangenome curve of *S*. *argenteus* was not saturated, suggesting the existence of an open pangenome in *S*. *argenteus* (Supplementary Figure 5). This inference was further supported by the high prevalence of “cloud genes” (genes found in less than 15% of the strains), which corresponded to approximately 59% of the pangenome. Although the genomic homology and differentiation of *S*. *argenteus* with respect to *S*. *aureus* deserve long-term attention, the genetic diversity within *S*. *argenteus* itself is also a point of interest. As two of the most prevalent clusters within *S*. *argenteus*, C1 and C2 showed different distributions within isolated sources and might represent two different evolutionary directions of this species. One of the differences between these two clusters is in the number of accessory genes. The greater number of accessory genes in C2 than in C1 indicates that C1 may be specialized for a specific environment, while C2 may have a better capacity to adapt to different environments (Merhej et al., 2013; Weinert et al., 2015; Pang et al., 2019). This is also in accord with the source distributions of the two clusters: C1 strains are mainly found in human infections, while C2 strains are attributed to multiple sources. As a result, the VFs preferentially found in C1 (e.g., PVL) may promote the infective potential of corresponding strains (Otto, 2013). In contrast, the greater number of sucrose synthases found in C2 may be a strategy for the better utilization of carbon sources in diverse environments. However, the possibility of transmission of this bacterium between humans and the environment is worthy of attention.

Interestingly, our results showed that the geographical structure of *S*. *argenteus* is geographical distribution. The global distribution of this bacterium has been previously shown (McDonald et al., 2006; Thaipadungpanit et al., 2015; Zhang et al., 2017), and this study further suggested that *S*. *argenteus* strains stabilized in certain regions might adapt to special niches by horizontally acquiring particular elements. One type of region-specific element is the SCC*mec* element. SCC*mec* elements can transport determinants of resistance to antimicrobial agents, virulence determinants and other genes important for bacterial survival under stress conditions, and it was shown in this study that the strains isolated from Europe were more likely to carry this type of element than those isolated from Asia. This finding is in accord with previous studies, in which MRSA-related *S*. *argenteus* has never been found in Asia but has occasionally been reported in Europe (McDonald et al., 2006; Yeap et al., 2017; Tång Hallbäck et al., 2018; Wu et al., 2020). In addition, some plasmids were preferentially located in particular regions (Supplementary Figure 6). All of these findings reveal potential gene exchange between *S*. *argenteus* strains and local *S*. *aureus* strains.

In fact, significant geographical specialization of *S*. *argenteus* is observed in strains isolated in the East Asia region. Bacterial strains in this region include multiple environmental isolates (Supplementary Table 1) and harbor a number of specific genes associated with antibiotic resistance, quorum sensing and biofilm formation. In fact, most *S*. *argenteus* strains isolated in China have been reported to show a strong or moderate ability to produce biofilms (Wu et al., 2020). This remarkable capacity to form biofilms may also account for the broad distribution of biofilms among different environmental sources and their transmission between the environment and humans (Zhang et al., 2017; Wu et al., 2020). Considering the presence of antibiotic resistance-conferring plasmids and their spread in the environment, the potential risk of plasmid-mediated resistance from environmental *S*. *argenteus* sources deserves more attention.

In summary, we used a multiscale comparative genomic approach to provide insights into the diversity of *S*. *argenteus* species. Our results revealed that *S*. *argenteus* may show a high level of pathogenicity similar to that of *S*. *aureus*, and one significant driving force behind the evolutionary divergence of *S*. *argenteus* may be the geographical distribution because of that the panmobilome of this species is closely related to the environmental bacteria they contacting in a specific region. New high-throughput DNA sequencing technologies and analysis techniques will continue to improve our ability to track and distinguish this pathogen. Furthermore, with the increase in the *S*. *argenteus* genome, a more extensive analysis combining disciplines such as evolutionary biology, climate science, and phylogeography will provide new insights into the complex interactions between these populations and the variable ecological conditions of their surrounding environments during the process of diversification, which are aspects that are critical for understanding the basis of the mechanisms driving the evolution of this novel species and the initiation of geographic expansion, specialization and epidemic radiation.

## Materials and Methods

### Bacterial Strains

All genome sequences of *S*. *argenteus* were downloaded from National Centre for Biotechnology Information (NCBI) databases (up to 1 March 2020) (Supplementary Table 1). For comparative genomic analysis, we added 24 environmental strains isolated from China (Supplementary Table 1). These isolates were distinguished from *S*. *aureus* isolates by MLST and PCR confirmation. Each isolate was stored in a Microbank (Pro-Lab Diagnostics, Richmond Hill, ON, Canada) at −80°C and recovered when it was analyzed.

### Whole-Genome Sequencing and Assembly

Genomic DNA was extracted from *S*. *argenteus* strains using a genomic DNA extraction kit (Magen Biotech, Guangzhou, China) according to the manufacturer’s instructions. Each DNA sample was fragmented into 400-bp fragments by a Covaris M200 sonicator and prepared for sequencing with the Ion Plus Fragment Library Kit (Thermo Fisher Scientific Inc., MA, United States). Whole genomes were sequenced on the Life Ion S5 platform with an average coverage of 100×. Clean reads were used for *de novo* assembly with SPAdes v3.6.2 (Bankevich et al., 2012).

### Phylogenetic Analysis of *Staphylococcus argenteus* Based on Core Genome Single Nucleotide Polymorphisms

The core genome of the *S*. *argenteus* strains was produced using Harvest v1.1.2 with the MSHR1132 genome as a reference (Treangen et al., 2014). After core genome alignment was performed, Gubbins was used to conduct recombination analysis and remove putative recombined regions (Croucher et al., 2015). SNPs were then extracted from the recombination-free core genome alignment using the script available at https://github.com/sanger-pathogens/snp-sites. The ML phylogenetic tree was constructed from the concatenated core SNPs using RAxML v8.2.10 with the GTRGAMMA model (1,000 bootstrap replicates) (Stamatakis, 2014) and was visualized using iTOL (Letunic and Bork, 2016).

Core genome SNP alignment was also employed to estimate the genetic population structure using the hierBAPS module of the Bayesian analysis of population structure (BAPS) software program, which fits lineages to genome data using nested clustering (Cheng et al., 2013). The estimation used three independent interactions with 15, 30, and 45 clusters at levels 1–3 of the hierarchy.

### Pan-Genome Analysis

The genomes of all analyzed isolates were annotated using Prokka v1.11 (Seemann, 2014). The output of Prokka was employed to construct the pangenome using Roary v3.11.2, with a BLASTP identity cut-off of 90% (Page et al., 2015). A matrix of the absence (0) or presence (1) of all genes was generated according to the pangenome distribution of all samples, and this matrix was used for subsequent analysis.

### Functional Classification

The functional annotation of Clusters of Orthologous Groups (COG) was performed using eggNOG software (Huerta-Cepas et al., 2019). The presence of VFs and antibiotic resistance factors encoded in the genomes was inferred by comparing all the proteins against the virulence factor database (VFDB; Chen et al., 2016), the comprehensive antibiotic resistance database (CARD; Jia et al., 2017), and Resfinder (Zankari et al., 2012). Pathway annotations were obtained from the Kyoto Encyclopedia of Genes and Genomes (KEGG) using the Automatic Annotation Server.

### Analysis of the Panmobilome

The SCC*mec* elements and plasmids in each genome sequence were detected using the online services SCC*mec*Finder 1.2^1^ and PlasmidFinder 2.1.^2^ The phages of each *S*. *argenteus* strain were detected using PhiSpy (Akhter et al., 2012).

## Data Availability Statement

The data presented in the study are deposited in the NCBI database under BioProject PRJNA769826.

## Author Contributions

QWu, JZ, SW, and RP conceived and designed the experiments. RP, JH, and FZ performed the experiments. SW, RP, and LX analyzed the data. QG, MC, ZC, QWa, YD, and JW contributed the reagents, materials, and analysis tools. SW, RP, and QWu contributed to wrote the manuscript. All authors contributed to the article and approved the submitted version.

## Conflict of Interest

ZC and QWa are employed by Guangdong Huankai Microbial Science and Technology Co. Ltd. The remaining authors declare that the research was conducted in the absence of any commercial or financial relationships that could be construed as a potential conflict of interest.

## Publisher’s Note

All claims expressed in this article are solely those of the authors and do not necessarily represent those of their affiliated organizations, or those of the publisher, the editors and the reviewers. Any product that may be evaluated in this article, or claim that may be made by its manufacturer, is not guaranteed or endorsed by the publisher.

## Footnotes

1
https://cge.cbs.dtu.dk/services/SCCmecFinder/

2
https://cge.cbs.dtu.dk/services/PlasmidFinder/

## Supplementary Material

Supplementary Figure 1 Bayesian analysis of the population structure of 153 *S. argenteus* strains. Each row in the figure represents a strain, each column represents an SNP, and horizontal black lines are drawn to separate the two clusters. The colour pattern of these SNPs within the black lines is consistent with their assignment to the same BAPS group.

Supplementary Figure 2 Hierarchical clustering in two dimensions of pairwise average nucleotide identity (ANI) comparisons of 153 *S. argenteus* strains. The ANI values are presented in a heatmap generated according to the matrix of the percentage identity.

Supplementary Figure 3 Maximum-likelihood tree of *S. argenteus* constructed from core genome single-nucleotide polymorphisms. The clade colours indicate the location of each strain. The bar colour indicates the BAPS cluster to which each strain is attributed.

Supplementary Figure 4 Maximum-likelihood (ML) tree of *S. argenteus* constructed from the full alignment of core genes.

Supplementary Figure 5 Pangenome features of 153 analysed *S. argenteus* strains. The dotted line indicates the number of novel genes discovered with the sequential addition of new genomes. The solid line indicates the values of the core genes as genomes are added to the pangenome.

Supplementary Figure 6 The number of predicted prophages in 153 *S. argenteus* strains. The clade colours indicate the source of each strain. The bar colour indicates the BAPS cluster to which each strain is attributed.

Supplementary Figure 7 KEGG pathway annotation of East Asia-specific genes in *S. argenteus*. **(A)** Beta-Lactam resistance pathway. **(B)** Vancomycin resistance pathway. **(C)** S. aureus infection pathway. **(D)** Quorum-sensing pathway. **(E)** Biofilm formation pathway. The green boxes indicate the specific genes.

Supplementary Table 1 *Staphylococcus argenteus* strains analysed in this study.

Supplementary Table 2 Gene information for the pangenome of *S. argenteus*.

Supplementary Table 3 Information on virulence factors in the *S. argenteus* pangenome.

Supplementary Table 4 Information on antibiotic resistance genes in the *S. argenteus* pangenome.

Supplementary Table 5 Information on region-specific genes of *S. argenteus*.
