# Supplementary figures and images for: Evolutionary Divergence of the Novel Staphylococcal Species Staphylococcus argenteus
Source: Front Microbiol. 2021 Nov 19;12:769642. doi: 10.3389/fmicb.2021.769642 (PMC8640356; doi:10.3389/fmicb.2021.769642)

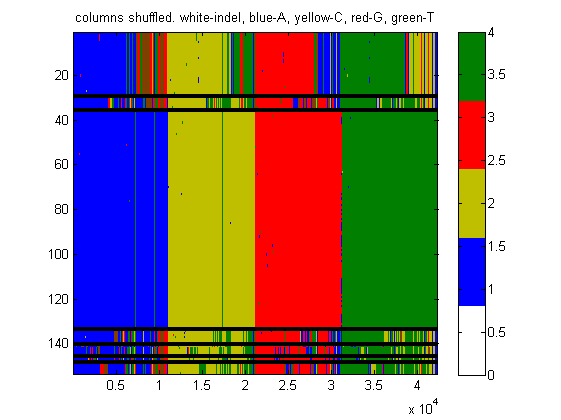

Supplement: Supplementary Figure 1 — Bayesian analysis of the population structure of 153 S. argenteus strains. Each row in the figure represents a strain, each column represents an SNP, and horizontal black lines are drawn to separate the two clusters. The colour pattern of these SNPs within the black lines is consistent with their assignment to the same BAPS group. [file Image_1.tif]

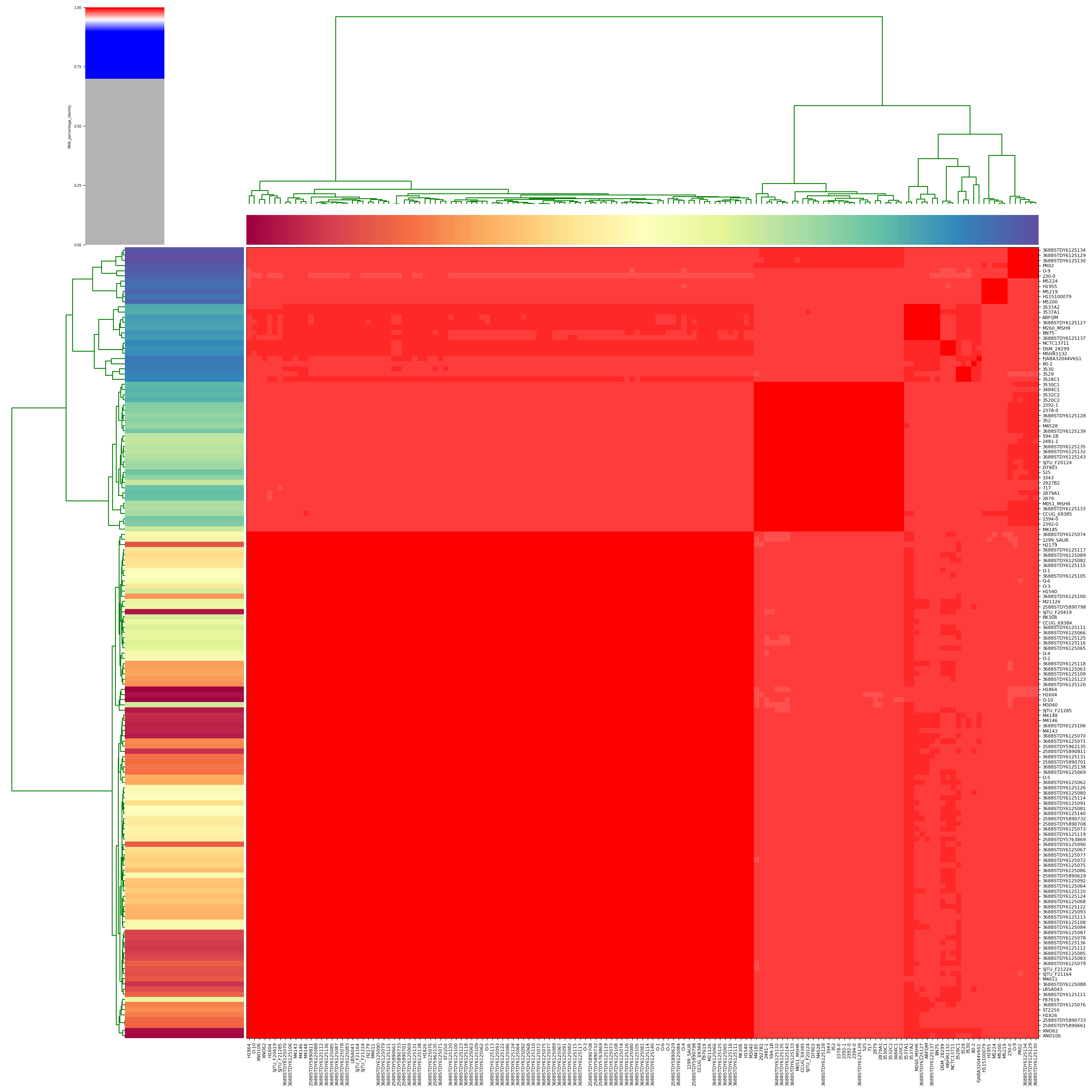

Supplement: Supplementary Figure 2 — Hierarchical clustering in two dimensions of pairwise average nucleotide identity (ANI) comparisons of 153 S. argenteus strains. The ANI values are presented in a heatmap generated according to the matrix of the percentage identity. [file Image_2.png]

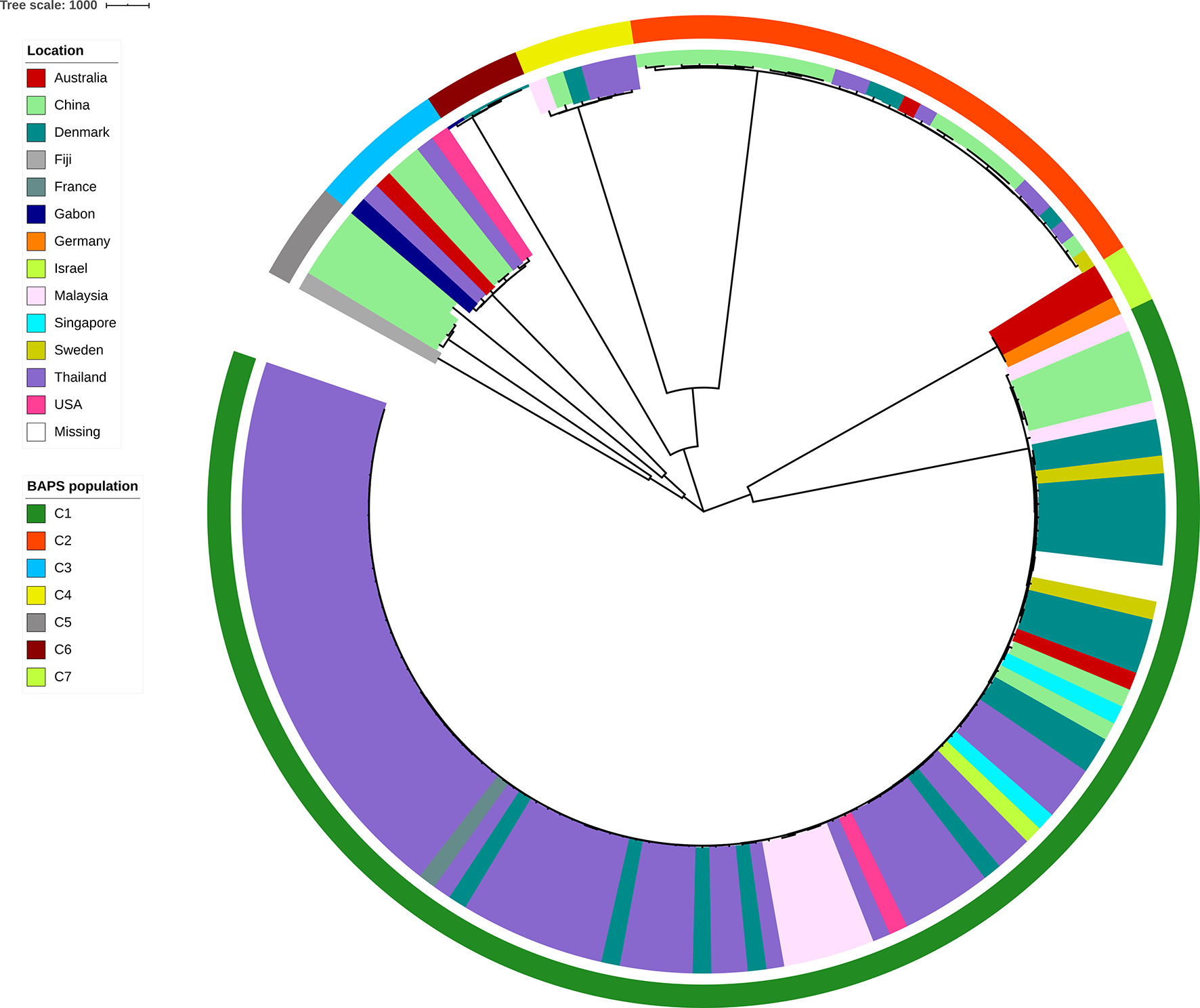

Supplement: Supplementary Figure 3 — Maximum-likelihood tree of S. argenteus constructed from core genome single-nucleotide polymorphisms. The clade colours indicate the location of each strain. The bar colour indicates the BAPS cluster to which each strain is attributed. [file Image_3.png]

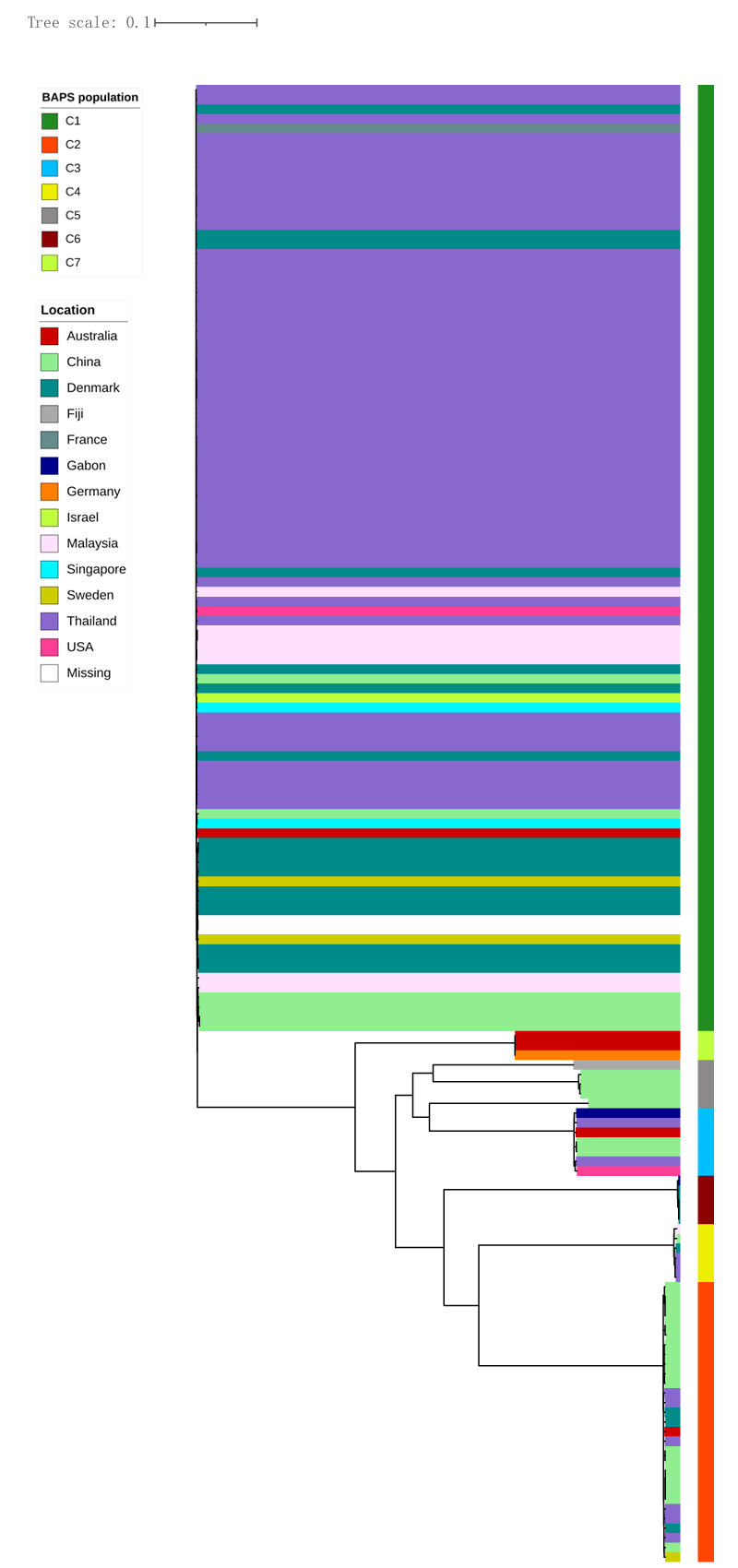

Supplement: Supplementary Figure 4 — Maximum-likelihood (ML) tree of S. argenteus constructed from the full alignment of core genes. [file Image_4.tif]

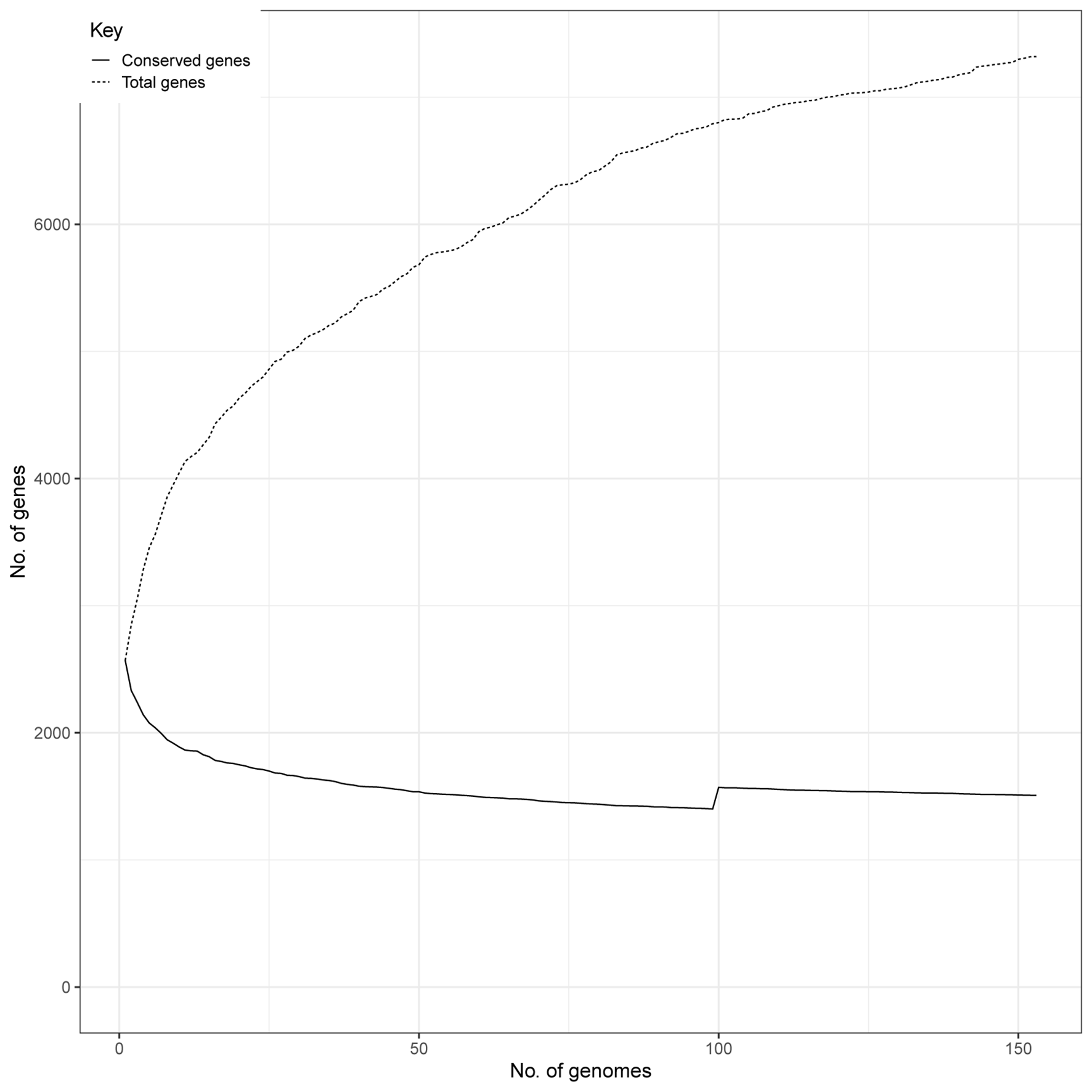

Supplement: Supplementary Figure 5 — Pangenome features of 153 analysed S. argenteus strains. The dotted line indicates the number of novel genes discovered with the sequential addition of new genomes. The solid line indicates the values of the core genes as genomes are added to the pangenome. [file Image_5.png]

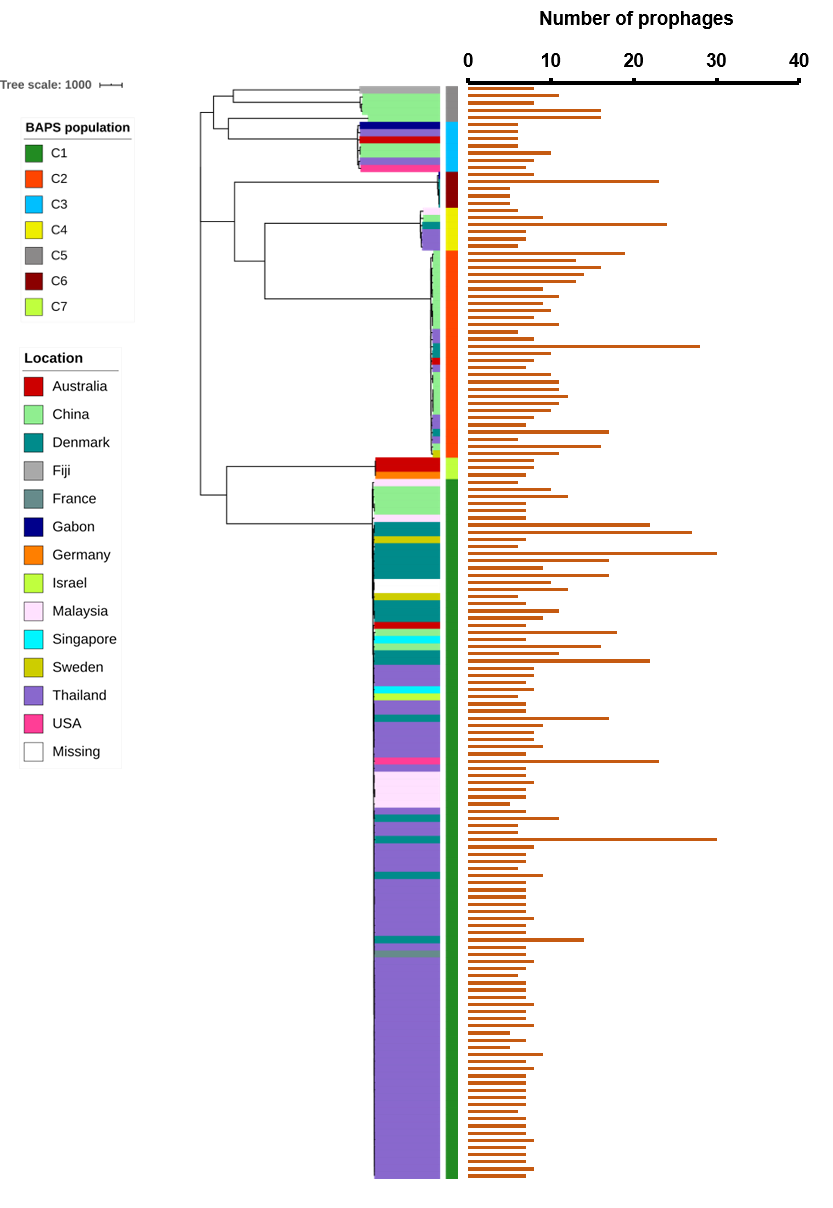

Supplement: Supplementary Figure 6 — The number of predicted prophages in 153 S. argenteus strains. The clade colours indicate the source of each strain. The bar colour indicates the BAPS cluster to which each strain is attributed. [file Image_6.tif]

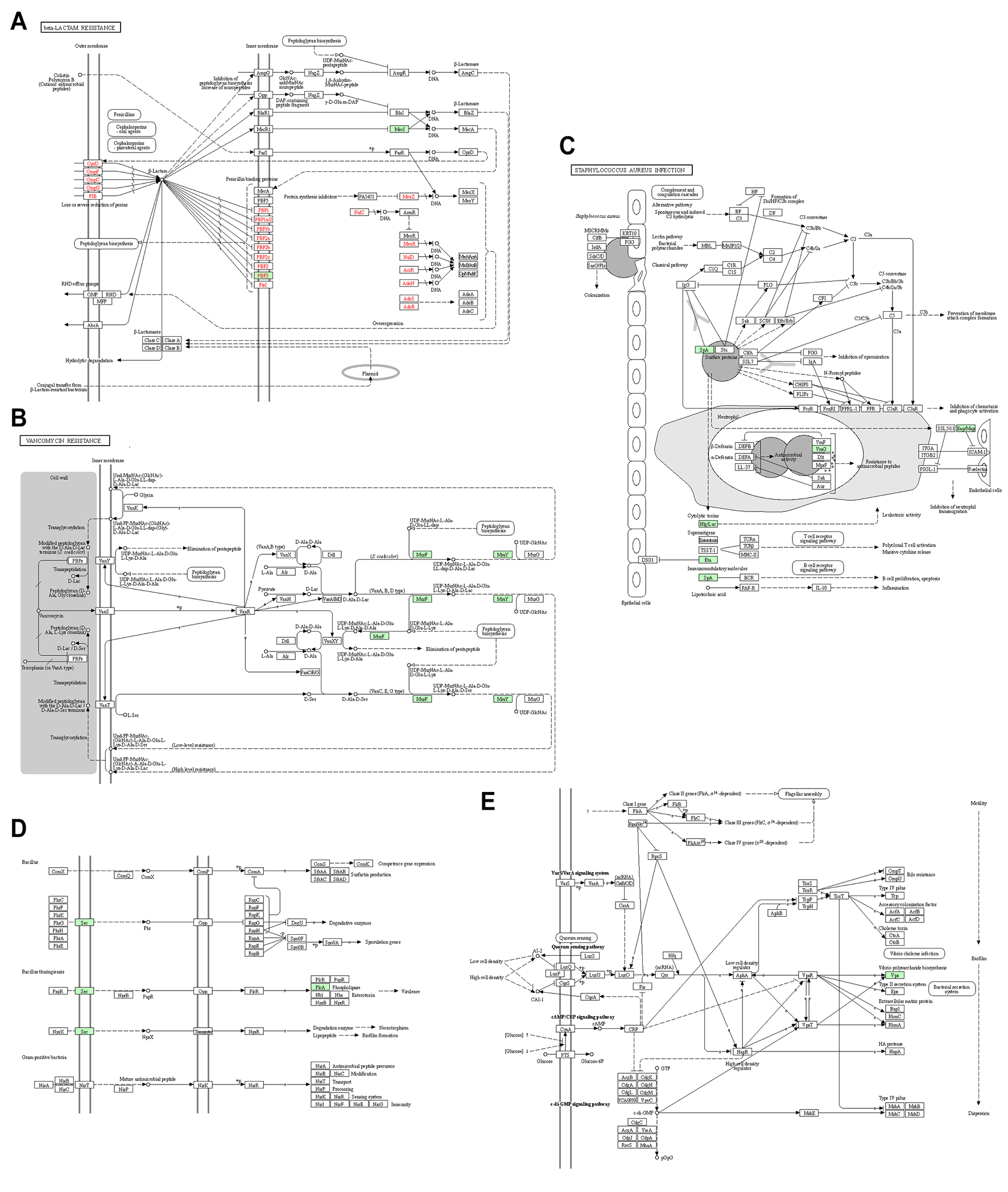

Supplement: Supplementary Figure 7 — KEGG pathway annotation of East Asia-specific genes in S. argenteus. (A) Beta-Lactam resistance pathway. (B) Vancomycin resistance pathway. (C) S. aureus infection pathway. (D) Quorum-sensing pathway. (E) Biofilm formation pathway. The green boxes indicate the specific genes. [file Image_7.tif]
